# A Case of Incidental Giant Intraductal Papilloma Containing Ductal Carcinoma In Situ

**DOI:** 10.1002/cnr2.70184

**Published:** 2025-03-18

**Authors:** Micaela Resta, Stephanie Fine

**Affiliations:** ^1^ University of New Mexico School of Medicine Albuquerque New Mexico USA; ^2^ Department of Surgery University of New Mexico Health Sciences Center Albuquerque New Mexico USA

**Keywords:** breast reconstruction, breast‐conserving surgery, DCIS, intraductal papilloma

## Abstract

**Background:**

Intraductal papillomas of the breast (IDPs) are typically small benign lesions characterized by pathologic discharge, a palpable mass, or an occult presentation. Giant IDPs (> 5 cm) are a rare entity. The few existing cases of giant IDPs describe grossly apparent exam findings and concerning features on imaging. While surgical excision is the standard of care for most IDPs, there is a scarcity of evidence to guide management of giant papillary tumors.

**Case:**

We describe a case of a 64‐year‐old female presenting with pathologic nipple discharge and no discrete palpable breast mass who was routinely diagnosed with ductal carcinoma in situ (DCIS). Imaging findings of mass calcifications and intraductal mass debris correlated with biopsy results, and the patient underwent central lumpectomy with oncoplastic reconstruction. At the time of surgery, the DCIS was incidentally found to be contained within a large solitary papilloma. The specimen pathology showed DCIS spanning a large area in a multifocal pattern that involved a 7 cm giant intraductal papilloma. Though margins were negative for malignancy, the benign papillary lesion was incompletely excised. A multidisciplinary review opted to pursue whole breast radiation therapy followed by adjuvant endocrine treatment over re‐excision.

**Conclusion:**

This unusual case highlights the potential for giant IDP to remain clinically occult in the background of known ductal carcinoma in situ and emphasizes the value of multidisciplinary care discussion and a patient‐centered approach to surgical decision making in the absence of existing guidelines.

## 1 Introduction

Intraductal papilloma (IDP) of the breast is a relatively common benign neoplasm, constituting about 5% of proliferative benign breast lesions, and is part of a heterogeneous group of papillary breast lesions [1, 2, 3]. Variability in the clinical and radiologic presentation of IDP makes preoperative characterization of these lesions challenging. Due to the correlation of IDP with breast cancer, as well as frequently overlapping symptoms between the two entities, differentiating benign from malignant features is a critical part of management. This distinction is further complicated in the rare case of large IDP lesions. In contrast to the small sizes typically seen in IDP, giant intraductal papillomas (> 5 cm) are far less common; we found fewer than 10 cases of giant IDP reported in existing literature, which nearly unanimously describe a large breast mass or concerning imaging features [3, 4, 5, 6, 7, 8]. Even fewer cases can be found for giant IDPs harboring carcinoma—a scenario more often described in multiple intraductal papillomas than in solitary IDPs [2]. Existing literature on these topics reviews the difficulties associated with preoperative identification of malignancy within such suspicious breast masses. In contrast, we present a case of ductal carcinoma in situ (DCIS) incidentally found to be contained by a giant solitary IDP at the time of surgical excision. This case report illustrates a unique presentation of giant IDP lacking a dominant palpable mass or any preoperative evidence to suggest this rare benign breast lesion as the underlying diagnosis during routine management of breast cancer. Additionally, we highlight the importance of multidisciplinary review.

## 2 Case Description

A 64‐year‐old female was referred to our clinic at the University of New Mexico Cancer Center in 2023 for evaluation of biopsy‐proven DCIS of the right breast. The patient initially had presented to an outside facility with 2 months of clear spontaneous right nipple discharge. She denied any noticeable breast mass or skin changes on self‐examination. Relevant history included a remote left breast lumpectomy for benign intraductal papilloma with atypical ductal hyperplasia and bilateral breast augmentation. Notably, the patient had been taking hormone replacement therapy for the past 10 years, initially prescribed by an outside provider for vasomotor symptoms of menopause, and discontinued at the time of her DCIS diagnosis. This regimen included estrogen, progestin, and testosterone, though details on formulation and dosages were limited by available records. Clinical examination of the right breast was significant for a vague, soft thickening between 12 and 3 o'clock measuring 3.5 × 2 cm, 1–2 cm from the nipple areolar complex. However, no discrete or dominant mass could be identified. Unilateral clear serous fluid was noted to arise spontaneously from a single central right nipple duct and increased in volume with palpation of the area of thickening.

**FIGURE 1:**
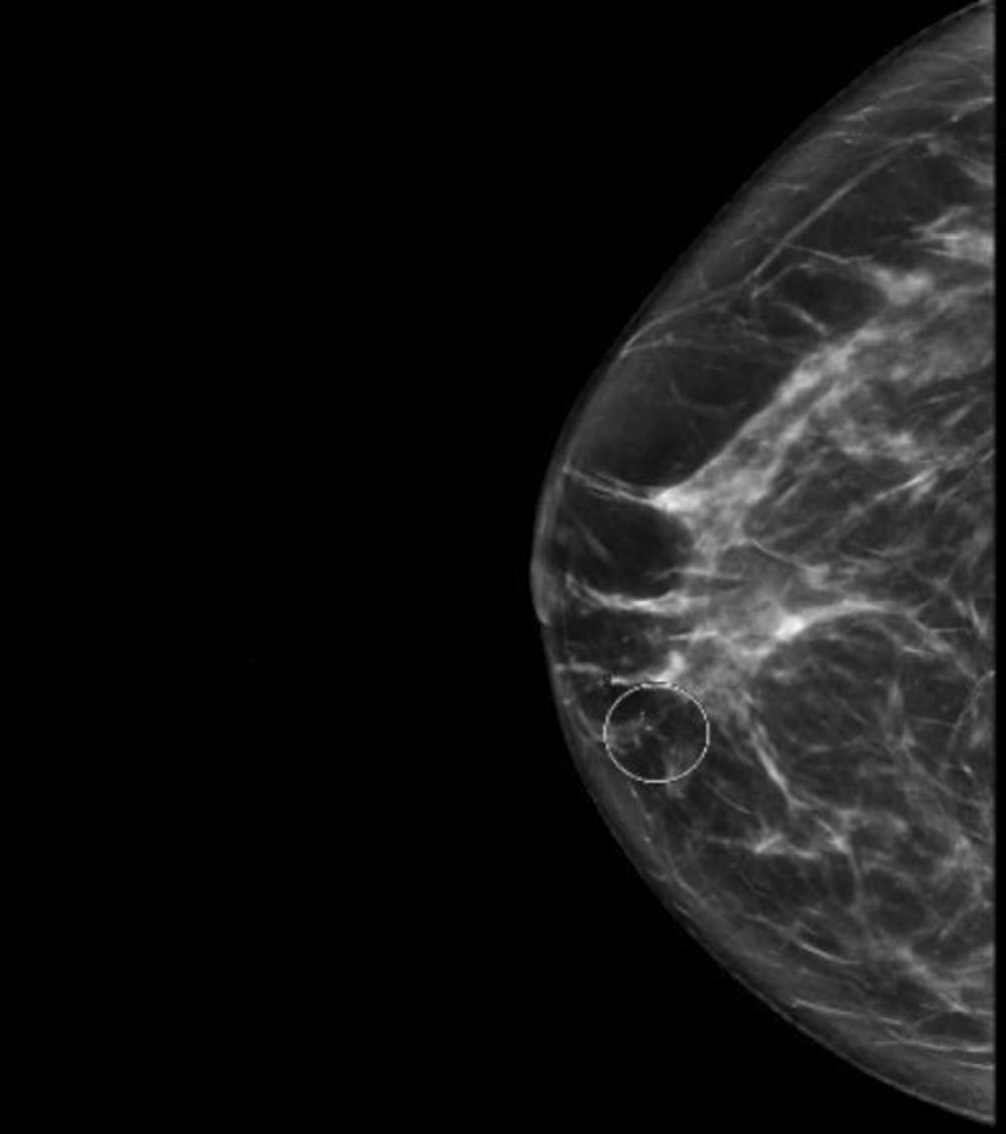
Diagnostic mammogram, unilateral, right breast craniocaudal view, with microcalcifications in the area of suspicion at 1 o'clock (circled).

**FIGURE 2:**
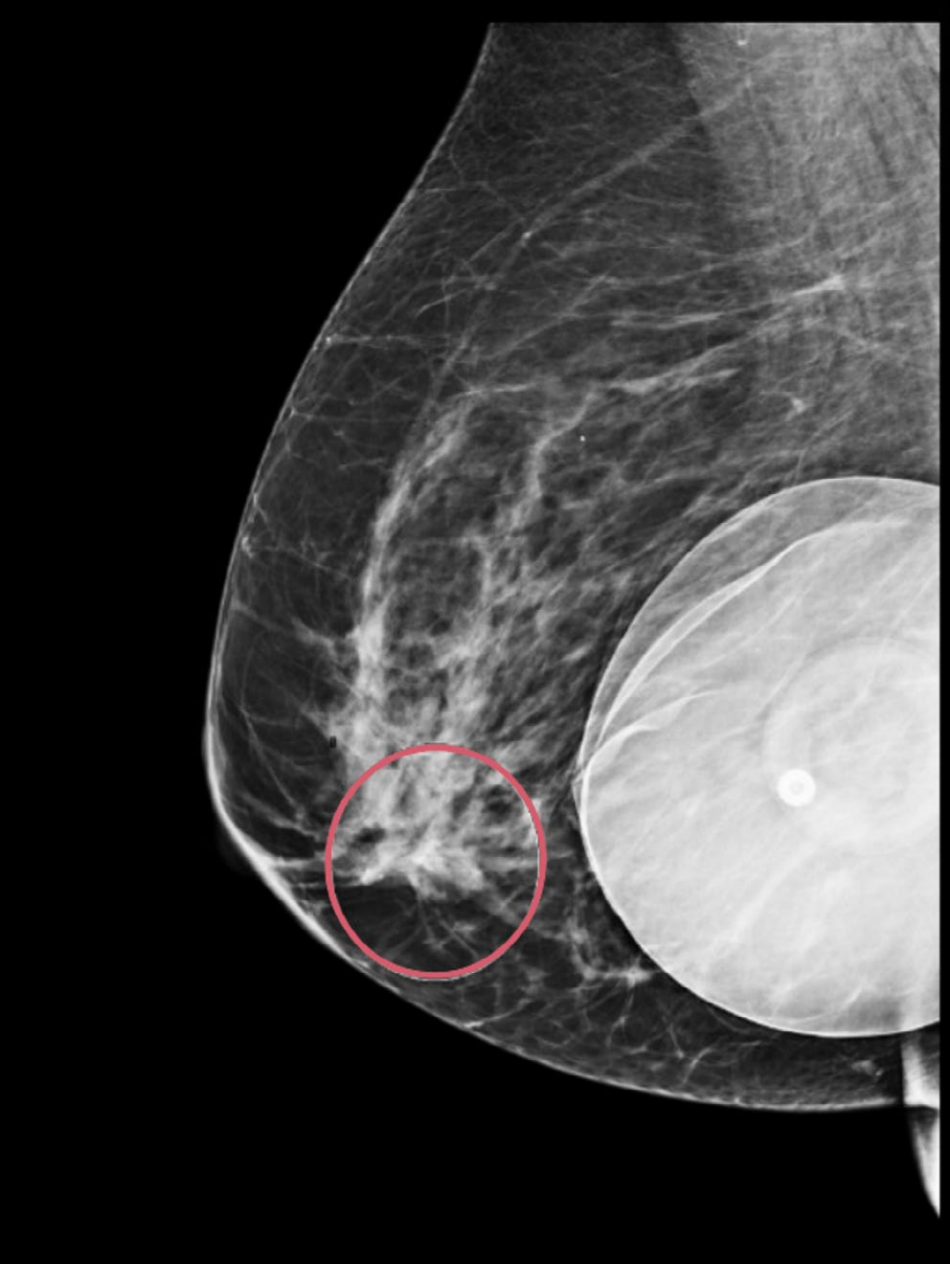
Diagnostic mammogram, unilateral, right breast mediolateral oblique view, nonimplant displaced, revealing the area of architectural distortion in the retroareolar central breast (circled) in relation to the intact retropectoral implant posteriorly.

**FIGURE 3:**
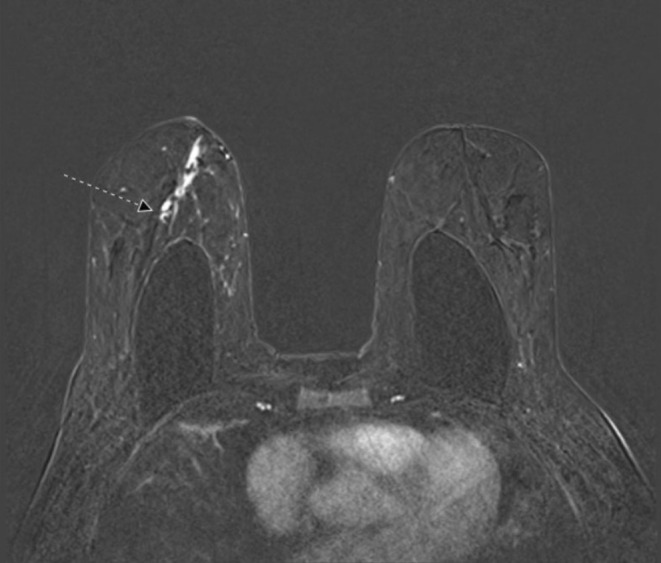
Breast MRI, bilateral, subtracted view, showing the 3.3 cm area of clumped and linear nonmass enhancement in the right medial, central, and retroareolar region, 0.9 cm from the base of the nipple and approaching the anterior aspect of the intact retropectoral implant (arrow).

Initial diagnostic mammography demonstrated suspicious right medial breast calcifications with possible right anterior asymmetry (Figure 1). Intraductal mass debris measuring 0.3 cm in the setting of retroareolar duct ectasia was also noted on mammography, which was benign in appearance and suspected to be the source of the patient's discharge (Figure 2). She subsequently underwent ultrasound‐guided biopsy of the suspicious area, revealing grade 2 ductal carcinoma in situ; microcalcifications were present in the DCIS, and ER/PR were both positive at > 90%. Further characterization of the lesion was pursued with MRI, which showed a 3.3 cm area of clumped and linear nonmass enhancement in the right medial, central, and retroareolar regions associated with multiple adjacent ducts containing proteinaceous blood and debris (Figure 3). This abnormal nonmass enhancement spanned greater than the area of mammographically identified calcifications, contacting the implant capsule posteriorly and coming within 0.9 cm of the nipple base anteriorly. The area of nonmass enhancement was interpreted as noncalcified DCIS. No other abnormalities were noted during workup, including on routine blood work.

Given proximity to the base of the nipple, surgical excision with either central lumpectomy or mastectomy with or without reconstruction was considered. The patient opted for breast conservation with central lumpectomy as well as bilateral implant removal. The plastic surgical service was enlisted to assist with the reconstructive challenges associated with nipple excision, large implant size, and relatively small breast tissue volume. Right central lumpectomy with bilateral removal of implants and oncoplastic reduction was performed. Excisional pathology was negative for invasive carcinoma but revealed DCIS involving a large intraductal papilloma in a multifocal fashion (the largest contiguous focus of DCIS measured 5 mm). The papilloma lesion was described as encompassing many branch ducts over an area measuring 7 cm. Clear margins of 3 mm or greater were achieved for the DCIS; however, the IDP was incompletely excised, extending to the superior and posterior margins. Biopsied sentinel and nonsentinel lymph nodes were negative for malignancy. Given that the previous biopsy had stained ER/PR positive, hormone receptor testing was not repeated on the surgical specimen. No additional biochemical investigations were conducted by pathology.

**FIGURE 4:**
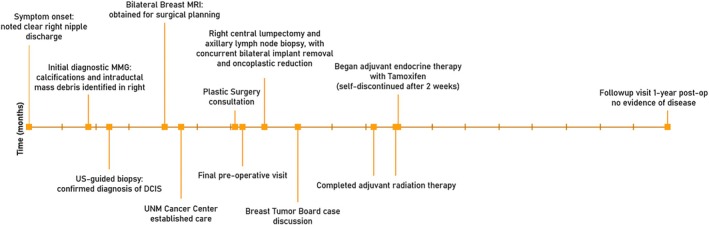
Clinical timeline of events, from symptom onset to most recent follow‐up visit. Horizontal axis corresponds to time in months, with tick marks at one‐month intervals. MMG = mammogram; MRI = magnetic resonance imaging; UNM = University of New Mexico; US = ultrasound.

Considering the incomplete resection of the papilloma, the case was discussed at the Breast Tumor Board with the consensus of proceeding with whole breast radiation therapy followed by adjuvant endocrine treatment, in line with consensus guidelines for DCIS management [9, 10]. The patient agreed to complete right breast radiation therapy using a protocol of 40.05 Gy dose/15 fraction 3D‐CRT, administered over a course of 21 days. Given the patient's concern for worsening of her baseline postmenopausal vasomotor symptoms with hormonal blockade therapy, a low‐dose regimen of endocrine therapy was chosen using tamoxifen (5 mg/day) for a planned duration of 5 years. However, the patient opted to discontinue tamoxifen after 2 weeks of use due to side effects of hot flashes and fatigue, and has continued to decline further treatment options. Ongoing follow‐up includes clinical examination every 6 months and high‐risk surveillance imaging with MRI and mammography annually [11]. No evidence of disease was found at our most recent follow‐up visit, 1 year out from surgery (Figure 4).

## 3 Discussion

IDP is the most common entity on the spectrum of papillary breast lesions and can be subdivided into its two common presentations: solitary central papillomas and multiple peripheral papillomas. Both IDP types share the same microscopic architecture of a papillary, arborescent growth pattern and overlying benign epithelia and myoepithelial, supported by a fibrovascular stalk; however, solitary and multiple IDP lesions are distinct in their clinical behavior, location, and malignancy risk [4, 12]. Multiple IDP arises peripherally from the terminal ductal lobular units. The location and small size of these lesions lend to an occult presentation, with a small palpable mass and pathologic discharge seen in less than 20% of cases [12]. Of the two types, multiple IDP is more closely associated with concurrent or subsequent breast carcinoma and has been reported to confer a 7.4‐fold increase in lifetime relative risk of developing breast cancer [12]. In comparison, solitary IDP arises centrally from the major lactiferous ducts and is more often associated with pathologic nipple discharge due to the retroareolar location. The risk of breast cancer development in solitary IDPs is minimal, as summarized by Tay et al. in a review of current IDP literature [13]. When compared to atypical ductal hyperplasia (ADH) or atypical lobular hyperplasia (ALH) of the breast parenchyma, multiple papilloma with ADH/ALH was associated with a higher relative risk of breast cancer whereas the risk associated with solitary papilloma with ADH/ALH was similar. The authors conclude that the solitary papilloma itself does not further increase the risk already attributed to atypical hyperplasia and highlight the significance of epithelial cell atypia and multiple type IDP as key prognostic factors [13]. In recent literature, there have been increasing efforts to identify predictive factors of IDP upgrading, with the most evidence supporting histologic atypia, multiple papilloma, radiologic calcification, and large lesion size (> 1 cm) [14]. There is little evidence that the location of the papilloma (arising centrally or peripherally) alone confers an independent association with the risk of malignancy; rather, the prognostic significance of central vs. peripheral papillomas is linked to their association with solitary and multiple tumor types, respectively. While the association between malignancy and IDP has been established, the origin of carcinoma and premalignant potential of these lesions is still debated.

The majority of IDPs are less than 2 cm in size and present with no clinical manifestations prior to imaging detection [5]. Giant IDPs are defined as measuring > 5 cm and are rarely reported. Among these cases available in the English literature, the presence of nipple discharge was variable, but a dominant, discretely palpable mass was unanimously reported [3, 4, 5, 6, 7, 8]. The largest of these masses reaches 15 cm in size and demonstrates visually apparent features on exam, ranging from a large elastic mass with a smooth surface as described by Kihara [6] to a multilobulated mass markedly distorting breast shape as described by Roy [7]. At such extreme sizes, IDPs are frequently complicated by secretion, debris, and hemorrhage following duct obstruction, leading to the formation of large cystic portions containing a relatively small neoplasm (intracystic papilloma). The development of mixed solid and cystic components likely accounts for the greater size and clinically apparent features of giant IDPs that are described in the literature. The lack of mixed solid and cystic areas by imaging could account for the lack of a clinically discrete mass in this case.

In general, no pathognomonic appearance for IDPs on radiology has been described. While IDPs are often clinically occult on mammography, they may be identified by clustered calcifications, small nodules, or atypical densities [5]. Ultrasound is more commonly employed for visualizing ductal dilation and intraductal or intracystic nodules. While less frequently ordered, MRI can provide further characterization of complicated lesions, as seen in most cases of giant IDP [3, 4, 5, 6, 7, 8]. Classically benign features of a small intraductal nodule, ductal dilation, well‐circumscribed mass, and smooth margins on MRI have been shown to be predictive of benign IDPs [15]. However, both benign and malignant IDPs are known to present with less reassuring morphology, including nodules without apparent ductal dilation, nonmass‐like enhancement of ductal pattern as in this case, isolated ductal dilation, and masses containing mixed solid and cystic components [16]. To our knowledge, all described giant IDPs share a radiologic appearance of mixed solid and cystic components which, in combination with an unusually large size, is highly concerning for malignancy [3, 4, 5, 6, 7, 8]. Not uncommonly, discordance between these concerning features and benign pathology on biopsy leads to prolonged diagnostic testing, misdiagnosis of carcinoma, and surgical excision of otherwise benign lesions. In contrast, the present case demonstrates early identification of DCIS secondary to mammographic calcifications. In the context of this lesion, the nonmass enhancing area seen on MRI was interpreted as noncalcified ductal carcinoma in situ rather than a separate entity. As with the more commonly described scenario of suspicious appearing benign lesions, described as “sheep in a wolf skin” [15], the indistinct characteristics of IDP on imaging complicated the distinction of this benign underlying lesion from the known carcinoma.

Existing guidelines have clearly established the role of radiation and endocrine therapy following breast‐conserving surgery for reducing recurrence rates in DCIS [9, 10, 11]. However, current management of breast papilloma is less clearly defined, with differing recommendations for excision versus imaging surveillance and clinical follow‐up. This is largely due to a lack of high predictive value among available diagnostic tests and variability in IDP upgrade rates. Some studies have attempted to define predictive factors for IDP upstaging, with patient age, a clinically palpable mass, lesion size, and calcification most consistently identified. Standardized interpretation models, such as Gottingen scores and Kinkel and Tozaki flowchart tools, have been shown to reliably characterize solitary IDPs as benign based on MRI appearance [15], though their utility in IDP detection and differentiating malignant from benign lesions has not been demonstrated. Atypical cytology is another frequently examined feature in predicting upgrade rates, with one study reporting IDP core biopsy findings of atypical ductal hyperplasia to be associated with malignancy at excision in as high as 67% of cases [17]. Despite most known cases reporting no atypia or DCIS on excisional pathology, outcome data for giant IDP is limited by its rarity. No studies are available on the efficacy of radiotherapy or endocrine therapy in reducing the risk of breast cancer in this setting.

With the paucity of evidence guiding management of benign giant papillary tumors, the unanticipated finding of an incompletely resected giant IDP in this case presents a unique clinical challenge. Consensus guidelines regarding breast conservation treatment for ER/PR positive DCIS involve adjuvant ipsilateral whole breast radiation therapy followed by the option of endocrine therapy with tamoxifen or an aromatase inhibitor in postmenopausal patients [9, 10]. However, the unique specimen pathology with DCIS spanning a large area in a “skip” pattern involving a giant intraductal papilloma required a more nuanced discussion. Of primary concern was the presence of papilloma at the superior and posterior lumpectomy margins that, in light of the skip nature of this lesion, raised the potential for residual carcinoma and recurrence despite achieving margins of greater than 3 mm. Multidisciplinary review focused on the value of attempting surgical re‐excision of IDP margins versus proceeding with standard layered adjuvant therapy. It concluded that the pursuit of clear margins for a benign papillary lesion would not provide a greater reduction in recurrence risk than would be achieved with whole breast radiation and adjuvant endocrine therapy. Furthermore, re‐excision would not affect the recommendation for whole breast radiotherapy. Therefore, the decision was made, using patient‐centered decision making, to proceed with whole breast radiation therapy using the previously described protocol. Following radiation, an endocrine therapy regimen of low‐dose tamoxifen (5 mg/day for 5 years) was chosen given this patient's concern regarding symptoms of hormone blockade therapy at standard dosing (20 mg/day) [9, 10].

Oncoplastic factors specific to this case posed a secondary challenge to treatment planning decisions. With a small baseline breast tissue volume following implant removal, the unusual specimen size and a larger‐than‐anticipated area necessitating excision left little remaining tissue for breast reconstruction. Aesthetic outcome was additionally complicated by the planned excision of the nipple areolar complex and excess breast skin secondary to overly large implants. The option of mastectomy with or without reconstruction was discussed during initial surgical planning and again postoperatively given the pathology findings. However, the patient ultimately desired breast conservation. Surgical planning was a joint effort of breast surgical oncology and plastic surgery services. Intentional rupture of saline implants was performed preoperatively to allow for skin envelope contraction, followed by breast‐conserving lumpectomy and bilateral oncoplastic reduction. Though the large tumor size resulted in mild breast asymmetry, a successful aesthetic outcome was achieved with this approach. Future surgical interventions to improve volume symmetry, including fat grafting, additional volume reduction, and repeat mastopexy, are options following radiation therapy completion.

## 4 Conclusion

Intraductal papillary lesions harbor the potential for breast carcinoma. While evidence continues to emerge regarding clinical, radiographic, and histologic factors predictive of upgrade rate, IDP lacks any pathognomonic appearance or reliable testing to inform management. Giant IDP is a rare entity often exhibiting concerning radiologic features that complicates clinical diagnosis. This unusual case describes the potential for giant IDP to remain clinically occult in the background of known ductal carcinoma in situ, with a resultant incomplete papilloma excision. Our patient's atypical presentation highlights the challenges in preoperative identification of this “lesion within a lesion” and the need for further studies to investigate the predictive value of diagnostic modalities. That said, the primary emphasis of this rare case is the importance of multidisciplinary care discussion and a patient‐centered approach to surgical decision making in the absence of existing literature to guide management.

## Author Contributions

All authors had full access to the data in the study and take responsibility for the integrity of the data and the accuracy of the data analysis. Conceptualization: Stephanie Fine. Methodology: Micaela Resta and Stephanie Fine. Resources: Stephanie Fine. Writing: Original Draft: Micaela Resta. Writing: Review and Editing: Micaela Resta and Stephanie Fine. Visualization: Micaela Resta; Supervision: Stephanie Fine.

## Ethics Statement

This study was submitted to the University of New Mexico Health Sciences Human Research Review Committee and determined to not involve human subjects research per the US Code of Federal Regulations policy 45 CFR 46.102(e) (1). Therefore, Institutional Review Board approval was not indicated. A statement of written informed consent has been obtained from the patient for the publication of case details and use of any images. All authors of this article certify that this work was conducted and submitted in accordance with the Committee on Publication Ethics and Wiley's Publication Ethics guidelines. All research was performed in an ethical and responsible manner without research misconduct, including data fabrication and falsification, plagiarism, image manipulation, unethical research, biased reporting, authorship abuse, redundant or duplicate publication, and undeclared conflicts of interest.

## Conflicts of Interest

The authors declare no conflicts of interest.

## Data Availability

Data sharing is not applicable to this article as no new data were created or analyzed in this study.
